# Supplementary material for: Modeling glioblastoma heterogeneity as a dynamic network of cell states
Source: Mol Syst Biol. 2021 Sep 16;17(9):e10105. doi: 10.15252/msb.202010105 (PMC8444284; doi:10.15252/msb.202010105)
Supplement: Supplementary file 6 — Source Data for Figure 5 [file MSB-17-e10105-s004.zip › Figure5A_sourcedata/GSEA_3017/nosigdb_stateF.GseaPreranked.1622795015644/VANGRONINGEN_MES.html]

Details for gene set VANGRONINGEN\_MES[GSEA]

|  || Dataset | state33017 |
| Phenotype | NoPhenotypeAvailable |
| Upregulated in class | na\_pos |
| GeneSet | VANGRONINGEN\_MES |
| Enrichment Score (ES) | 0.427944 |
| Normalized Enrichment Score (NES) | 2.8809934 |
| Nominal p-value | 0.0 |
| FDR q-value | 0.0 |
| FWER p-Value | 0.0 |
Table: GSEA Results Summary

  

Fig 1: Enrichment plot: VANGRONINGEN\_MES      
 Profile of the Running ES Score & Positions of GeneSet Members on the Rank Ordered List

  

| PROBE | GENE SYMBOL | GENE\_TITLE | RANK IN GENE LIST | RANK METRIC SCORE | RUNNING ES | CORE ENRICHMENT || 1 | GDF15 |  |  | 2 | 2.198 | 0.0308 | Yes |
| 2 | SPARCL1 |  |  | 6 | 1.804 | 0.0555 | Yes |
| 3 | APOE |  |  | 17 | 1.244 | 0.0688 | Yes |
| 4 | SDCBP |  |  | 27 | 1.054 | 0.0797 | Yes |
| 5 | LRRC17 |  |  | 31 | 1.044 | 0.0934 | Yes |
| 6 | DKK3 |  |  | 37 | 0.994 | 0.1054 | Yes |
| 7 | IFITM3 |  |  | 38 | 0.987 | 0.1197 | Yes |
| 8 | A2M |  |  | 42 | 0.974 | 0.1323 | Yes |
| 9 | SPARC |  |  | 52 | 0.929 | 0.1415 | Yes |
| 10 | NQO1 |  |  | 53 | 0.918 | 0.1548 | Yes |
| 11 | SQSTM1 |  |  | 54 | 0.914 | 0.1680 | Yes |
| 12 | PLK2 |  |  | 77 | 0.805 | 0.1692 | Yes |
| 13 | PON2 |  |  | 93 | 0.764 | 0.1731 | Yes |
| 14 | APP |  |  | 95 | 0.759 | 0.1836 | Yes |
| 15 | IFITM2 |  |  | 103 | 0.749 | 0.1911 | Yes |
| 16 | LTBP1 |  |  | 108 | 0.741 | 0.2000 | Yes |
| 17 | B2M |  |  | 110 | 0.736 | 0.2101 | Yes |
| 18 | NPC2 |  |  | 114 | 0.728 | 0.2192 | Yes |
| 19 | FILIP1L |  |  | 116 | 0.722 | 0.2292 | Yes |
| 20 | PHLDA3 |  |  | 131 | 0.696 | 0.2326 | Yes |
| 21 | FIBIN |  |  | 147 | 0.673 | 0.2353 | Yes |
| 22 | ITPR1 |  |  | 149 | 0.667 | 0.2444 | Yes |
| 23 | PTGFRN |  |  | 151 | 0.664 | 0.2536 | Yes |
| 24 | ITM2C |  |  | 160 | 0.649 | 0.2591 | Yes |
| 25 | ATP1B1 |  |  | 167 | 0.635 | 0.2655 | Yes |
| 26 | ATP2B1 |  |  | 170 | 0.632 | 0.2737 | Yes |
| 27 | ITM2B |  |  | 181 | 0.610 | 0.2778 | Yes |
| 28 | PLOD2 |  |  | 183 | 0.607 | 0.2861 | Yes |
| 29 | CD44 |  |  | 191 | 0.600 | 0.2914 | Yes |
| 30 | COL5A2 |  |  | 199 | 0.589 | 0.2966 | Yes |
| 31 | PDGFC |  |  | 219 | 0.577 | 0.2960 | Yes |
| 32 | HTRA1 |  |  | 229 | 0.568 | 0.2999 | Yes |
| 33 | LIFR |  |  | 239 | 0.562 | 0.3038 | Yes |
| 34 | GPR137B |  |  | 265 | 0.541 | 0.2998 | Yes |
| 35 | FAM3C |  |  | 266 | 0.541 | 0.3076 | Yes |
| 36 | CRISPLD1 |  |  | 268 | 0.538 | 0.3149 | Yes |
| 37 | ERBIN |  |  | 288 | 0.518 | 0.3134 | Yes |
| 38 | POSTN |  |  | 296 | 0.511 | 0.3174 | Yes |
| 39 | ATP6V0E1 |  |  | 303 | 0.510 | 0.3220 | Yes |
| 40 | TRIM5 |  |  | 305 | 0.508 | 0.3289 | Yes |
| 41 | HES1 |  |  | 320 | 0.501 | 0.3295 | Yes |
| 42 | IL6ST |  |  | 321 | 0.500 | 0.3367 | Yes |
| 43 | GPC6 |  |  | 332 | 0.495 | 0.3391 | Yes |
| 44 | ITGAV |  |  | 344 | 0.490 | 0.3410 | Yes |
| 45 | COL4A1 |  |  | 347 | 0.489 | 0.3471 | Yes |
| 46 | ATP2B4 |  |  | 356 | 0.485 | 0.3503 | Yes |
| 47 | MMP2 |  |  | 357 | 0.485 | 0.3573 | Yes |
| 48 | LIPA |  |  | 364 | 0.483 | 0.3615 | Yes |
| 49 | HSPA5 |  |  | 373 | 0.480 | 0.3646 | Yes |
| 50 | ADAM19 |  |  | 382 | 0.477 | 0.3677 | Yes |
| 51 | PRCP |  |  | 426 | 0.460 | 0.3540 | Yes |
| 52 | KDM5B |  |  | 439 | 0.455 | 0.3549 | Yes |
| 53 | LAMP1 |  |  | 440 | 0.455 | 0.3615 | Yes |
| 54 | SPRY4 |  |  | 443 | 0.453 | 0.3671 | Yes |
| 55 | PPIC |  |  | 462 | 0.445 | 0.3650 | Yes |
| 56 | SEC14L1 |  |  | 465 | 0.445 | 0.3705 | Yes |
| 57 | IGF2R |  |  | 470 | 0.444 | 0.3750 | Yes |
| 58 | GRN |  |  | 474 | 0.443 | 0.3800 | Yes |
| 59 | STK38L |  |  | 482 | 0.440 | 0.3831 | Yes |
| 60 | LRP10 |  |  | 492 | 0.438 | 0.3852 | Yes |
| 61 | GJA1 |  |  | 495 | 0.436 | 0.3905 | Yes |
| 62 | FZD7 |  |  | 504 | 0.434 | 0.3930 | Yes |
| 63 | PEA15 |  |  | 521 | 0.427 | 0.3916 | Yes |
| 64 | TFPI |  |  | 531 | 0.421 | 0.3934 | Yes |
| 65 | ANTXR1 |  |  | 537 | 0.418 | 0.3971 | Yes |
| 66 | IGFBP5 |  |  | 564 | 0.410 | 0.3907 | Yes |
| 67 | CNN3 |  |  | 576 | 0.407 | 0.3914 | Yes |
| 68 | RAB31 |  |  | 580 | 0.404 | 0.3958 | Yes |
| 69 | CTSO |  |  | 584 | 0.403 | 0.4002 | Yes |
| 70 | SDC2 |  |  | 596 | 0.399 | 0.4008 | Yes |
| 71 | NBR1 |  |  | 601 | 0.397 | 0.4046 | Yes |
| 72 | TNC |  |  | 613 | 0.393 | 0.4051 | Yes |
| 73 | SASH1 |  |  | 614 | 0.393 | 0.4108 | Yes |
| 74 | DPY19L1 |  |  | 673 | 0.378 | 0.3888 | Yes |
| 75 | EHD2 |  |  | 680 | 0.375 | 0.3914 | Yes |
| 76 | CCDC80 |  |  | 683 | 0.375 | 0.3958 | Yes |
| 77 | TNFRSF1A |  |  | 686 | 0.374 | 0.4003 | Yes |
| 78 | DNAJC3 |  |  | 689 | 0.374 | 0.4048 | Yes |
| 79 | SH3BGRL |  |  | 695 | 0.373 | 0.4078 | Yes |
| 80 | RAB29 |  |  | 706 | 0.369 | 0.4084 | Yes |
| 81 | ADAM9 |  |  | 722 | 0.365 | 0.4066 | Yes |
| 82 | COL27A1 |  |  | 760 | 0.356 | 0.3942 | Yes |
| 83 | RBMS1 |  |  | 761 | 0.355 | 0.3993 | Yes |
| 84 | FSTL1 |  |  | 774 | 0.352 | 0.3987 | Yes |
| 85 | SLC38A2 |  |  | 785 | 0.350 | 0.3991 | Yes |
| 86 | ID3 |  |  | 789 | 0.349 | 0.4027 | Yes |
| 87 | KCTD12 |  |  | 808 | 0.345 | 0.3992 | Yes |
| 88 | ACTA2 |  |  | 813 | 0.344 | 0.4022 | Yes |
| 89 | PLEKHH2 |  |  | 822 | 0.343 | 0.4034 | Yes |
| 90 | TMEM263 |  |  | 825 | 0.342 | 0.4074 | Yes |
| 91 | C1orf54 |  |  | 830 | 0.341 | 0.4104 | Yes |
| 92 | RGL1 |  |  | 831 | 0.341 | 0.4154 | Yes |
| 93 | RCN1 |  |  | 849 | 0.337 | 0.4122 | Yes |
| 94 | LAPTM4A |  |  | 854 | 0.335 | 0.4151 | Yes |
| 95 | COL6A2 |  |  | 855 | 0.335 | 0.4200 | Yes |
| 96 | RHOJ |  |  | 874 | 0.331 | 0.4162 | Yes |
| 97 | STAT3 |  |  | 880 | 0.330 | 0.4186 | Yes |
| 98 | L3HYPDH |  |  | 883 | 0.329 | 0.4224 | Yes |
| 99 | ARL4A |  |  | 889 | 0.328 | 0.4248 | Yes |
| 100 | CD59 |  |  | 897 | 0.326 | 0.4262 | Yes |
| 101 | RIT1 |  |  | 916 | 0.324 | 0.4224 | Yes |
| 102 | PEAK1 |  |  | 918 | 0.323 | 0.4266 | Yes |
| 103 | ATP10D |  |  | 938 | 0.320 | 0.4222 | Yes |
| 104 | TMBIM4 |  |  | 943 | 0.319 | 0.4249 | Yes |
| 105 | FAT1 |  |  | 968 | 0.315 | 0.4181 | Yes |
| 106 | RGS3 |  |  | 969 | 0.315 | 0.4227 | Yes |
| 107 | SCPEP1 |  |  | 971 | 0.314 | 0.4267 | Yes |
| 108 | HIBADH |  |  | 979 | 0.313 | 0.4279 | Yes |
| 109 | PLPP1 |  |  | 1017 | 0.307 | 0.4149 | No |
| 110 | SERPINE2 |  |  | 1030 | 0.304 | 0.4136 | No |
| 111 | SCRG1 |  |  | 1032 | 0.304 | 0.4175 | No |
| 112 | PLEKHA2 |  |  | 1113 | 0.290 | 0.3838 | No |
| 113 | SYPL1 |  |  | 1137 | 0.287 | 0.3771 | No |
| 114 | ATXN1 |  |  | 1138 | 0.286 | 0.3812 | No |
| 115 | DMD |  |  | 1153 | 0.285 | 0.3787 | No |
| 116 | WIPI1 |  |  | 1174 | 0.283 | 0.3733 | No |
| 117 | CTNNA1 |  |  | 1228 | 0.276 | 0.3522 | No |
| 118 | FN1 |  |  | 1238 | 0.275 | 0.3519 | No |
| 119 | CDH11 |  |  | 1240 | 0.275 | 0.3554 | No |
| 120 | PCSK5 |  |  | 1247 | 0.274 | 0.3566 | No |
| 121 | SGK1 |  |  | 1283 | 0.270 | 0.3439 | No |
| 122 | SSR1 |  |  | 1300 | 0.268 | 0.3402 | No |
| 123 | PRRX1 |  |  | 1341 | 0.262 | 0.3250 | No |
| 124 | NOTCH2 |  |  | 1420 | 0.255 | 0.2918 | No |
| 125 | EFEMP2 |  |  | 1428 | 0.255 | 0.2922 | No |
| 126 | CBFB |  |  | 1499 | -0.259 | 0.2628 | No |
| 127 | ANXA2 |  |  | 1511 | -0.263 | 0.2614 | No |
| 128 | ITGB1 |  |  | 1521 | -0.265 | 0.2609 | No |
| 129 | PHTF2 |  |  | 1529 | -0.267 | 0.2615 | No |
| 130 | ZFP36L1 |  |  | 1590 | -0.284 | 0.2372 | No |
| 131 | ENAH |  |  | 1592 | -0.284 | 0.2408 | No |
| 132 | EMP1 |  |  | 1598 | -0.285 | 0.2426 | No |
| 133 | PTBP1 |  |  | 1756 | -0.342 | 0.1732 | No |
| 134 | RAB13 |  |  | 1761 | -0.345 | 0.1763 | No |
| 135 | LGALS1 |  |  | 1799 | -0.362 | 0.1640 | No |
| 136 | NES |  |  | 1811 | -0.370 | 0.1641 | No |
| 137 | SEPT10 |  |  | 1888 | -0.414 | 0.1341 | No |
| 138 | TRAM2 |  |  | 1906 | -0.429 | 0.1323 | No |
| 139 | KDELR2 |  |  | 1925 | -0.439 | 0.1301 | No |
| 140 | TM4SF1 |  |  | 1948 | -0.452 | 0.1262 | No |
| 141 | TUBB6 |  |  | 2006 | -0.500 | 0.1064 | No |
| 142 | TNFRSF12A |  |  | 2116 | -0.694 | 0.0649 | No |
Table: GSEA details [plain text format]

  

Fig 2: VANGRONINGEN\_MES: Random ES distribution      
 Gene set null distribution of ES for **VANGRONINGEN\_MES**
